# Synthesis, Anticancer, Antimicrobial and Antioxidant Potential of Novel 4-(Substituted phenyl-1,3,4-oxadiazol/thiadiazol-2-yl)-4-(4-substituted phenyl) Azetidin-2-One Derivatives

**DOI:** 10.3390/ph16040517

**Published:** 2023-03-30

**Authors:** Davinder Kumar, Navidha Aggarwal, Virender Kumar, Harsh Kumar, Aakash Deep, Shabana Bibi, Hitesh Chopra, Rakesh Kumar Marwaha, Abdulrahman Alshammari, Metab Alharbi, Abdul Hayee

**Affiliations:** 1 Department of Pharmaceutical Sciences, Maharishi Dayanand University, Rohtak 124001, India; 2 MM College of Pharmacy, Maharishi Markandeshwar (Deemed to be University), Mullana 133207, India; 3 Department of Pharmaceutical Sciences, Chaudhary Bansi Lal University, Bhiwani 127021, India; 4 Department of Biosciences, Shifa Tameer-e-Millat University, Islamabad 41000, Pakistan; 5 Yunnan Herbal Laboratory, College of Ecology and Environmental Sciences, Yunnan University, Kunming 650091, China; 6 Chitkara College of Pharmacy, Chitkara University, Punjab 140401, India; 7 Department of Pharmacology and Toxicology, College of Pharmacy, King Saud University, Riyadh 11451, Saudi Arabia; 8 Department of Immunology, Faculty of Medicine, Academic Assembly, University of Toyama, Toyama 3190, Japan

**Keywords:** anticancer, MCF-7, 1,3,4-oxadiazole/thiadiazole, azetidin-2-one derivatives, antioxidant potential

## Abstract

By exploiting the ample biological potential of 1,3,4-oxadiazole/thiadiazole ring, 4-substitutedphenyl-1,3,4-oxadiazol/Thiadiazol-2-yl)-4-(4-substitutedphenyl) azetidin-2-one derivatives were prepared. Various substituted azetidin-2-one derivatives have been identified as immunostimulating and antimicrobial, as well as their antioxidant activity. 2-amino 1,3,4 oxadiazole/thiadiazole conjugates were synthesized by mixing semi/thio carbazides and sodium acetate with water and stirring well, followed by adding aldehydes in methanol at room temperature. Acetate (glacial) was used as the catalyst to produce Schiff’s bases (intermediates) by treating substituted aldehydes with 2-amino 1,3,4 oxadiazole/thiadiazole(s). Using the mixture of triethylamine (dropwise) and chloroacetylchloride with vigorous stirring, 4-substitutedphenyl-1,3,4-oxadiazol/Thiadiazol-2-yl)-4-(4-substitutedphenyl) azetidin-2-one derivatives were prepared. The newly synthesized conjugates were evaluated for their anticancer potential using MCF-7 cell lines. Amoxicillin and fluconazole were used as reference drugs to determine their antimicrobial activity. Synthesized derivatives were evaluated for their antioxidant properties using 2-diphenyl-1-picrylhydrazyl (DPPH). In vitro cytotoxicity screening (MTTS assay) revealed that derivatives AZ-5, 9, 10, 14 and 19 demonstrated high efficacy with the percentage of inhibition at different concentration ranges (0.1 μM, 0.5 μM, 1 μM, 2 μM) of 89% to 94% μM as compared to doxorubicin as standard drug. The antimicrobial study indicated that compounds AZ-10, 19, and AZ-20 were found to have significant antimicrobial potential with MIC ranges of 3.34 µM to 3.71 µM in comparison to reference drugs having 4.29 µM to 5.10 µM. Based on antioxidant screening, most of the synthetic derivatives showed greater stability and effectiveness than the standard drug. According to the antioxidant screening, compounds AZ-5 and AZ-15 (IC_50_ = 45.02 μg/mL and 42.88 μg/mL, respectively) showed the greatest potency, as compared to ascorbic acid (IC_50_ = 78.63 μg/mL). Structure-activity relationship (SAR) studies of synthesized novel derivatives revealed that para-substituted halogen and nitro derivatives have remarkable potential against MCF-7 cancer cell lines and different microbial strains. Current evidence indicates that the synthesized derivatives may be promising candidates for use in the prevention and treatment of these infections. These synthesized compounds require further mechanism-based research to understand how they interact with the cells.

## 1. Introduction

Cancer is an illness that can be fatal, and it is one of the leading causes of mortality worldwide. Breast cancer is one of the most common malignancies among women. Most breast cancer deaths are caused by metastasis, which is the primary cause of treatment failure. Breast cancer affects approximately 40 million women worldwide, mostly in regions with high human development indices [1,2].

**Figure 1:**
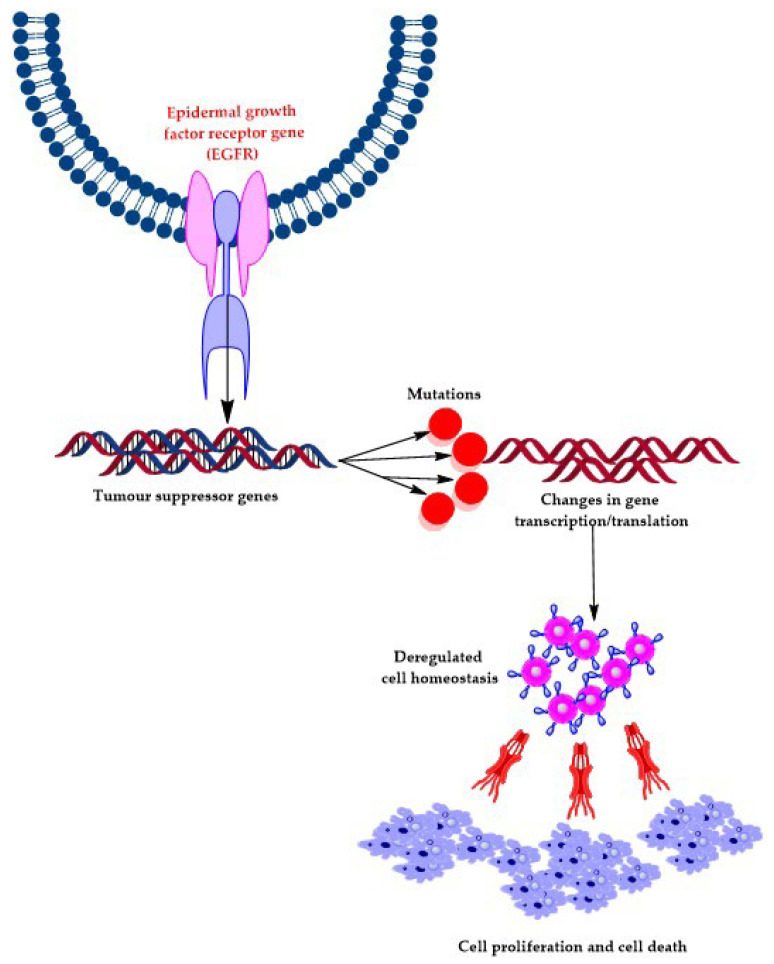
Mutations in EGFR cause breast cancer.

In breast cancer, tumor cells have the same characteristics and can be treated as if they were basal-like cells. Gene transcription and translation alterations can lead to functional defects that destabilize cell homeostasis and occur in oncogenes or tumor suppressor genes [3,4]. Mutation usually affects genes involved in the growth, proliferation, or death of cells (Figure 1). Detecting and treating the localized disease at an early stage generally results in 99% overall survival for patients with a low to intermediate risk of recurrence [5,6]. A lumpectomy or mastectomy can be performed to remove the tumor or the entire breast. Certain kinds of breast cancer may be treated with hormones through medications such as Tamoxifen and aromatase inhibitors. Breast cancer research relies heavily on the development and testing of novel drugs. Novel compounds, however, are often needed to fill this gap because the rate of discovery of new drugs is relatively slow. Thus, the search for new derivatives that can combat breast cancer is essential [7,8].

Investigations demonstrate that inflammatory factors can damage breast cells by releasing ROS and RNS with oxidative stress. This causes a pattern of cells to continually shift and experience distress. Cells can become cancerous as a result of the damage from free radicals [9,10,11,12]. To prevent cancer, antioxidants neutralize free radicals that can damage cells and make them cancerous. To ward off infection and inflammation in cells, new antimicrobial and antioxidant agents must be developed [13,14,15,16,17,18].

**Figure 2:**
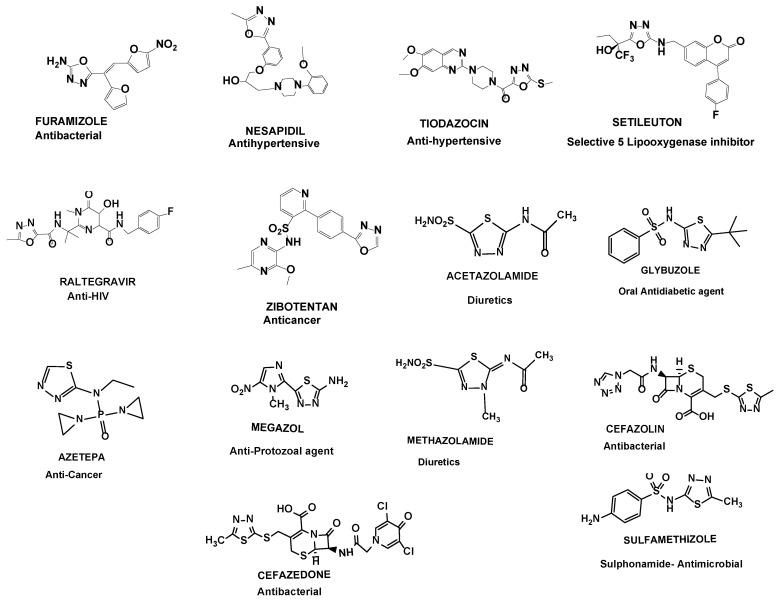
1,3,4-oxadiazole/thiadiazole drugs.

It is essential that a drug has specific and targeted pharmacological action in order to qualify as highly effective, since these actions can treat a variety of diseases, including cancer. Therefore, nitrogen, sulfur, and oxygen-containing heterocyclic scaffolds have attracted significant attention in the chemotherapeutic domain due to their importance in medicinal chemistry. Therefore, 1,3,4-oxadiazole/thiadiazole derivatives have wide-spectrum pharmacological actions. There have been some reports suggesting that a 1,3,4-oxadiazole/thiadiazole moiety may be effective as an anticancer [19,20,21], antimicrobial [22,23], antioxidant [24,25], tyrosinase inhibitor [26], cathepsin K inhibitor [27], anti-tubercular [28,29], anti-diabetics [30], anti-inflammatory [31,32,33], anticonvulsant [34,35], antihypertensive activities [36], etc. These heterocyclic scaffolds are the main structural backbone of a wide range of drugs. As such, these scaffolds occupy the focal point of many commercial drugs (Figure 2).

This research paper highlights the anti-breast cancer and antimicrobial potential of azetidin-2-one derivatives of 1,3,4-oxadiazole/thiadiazole ring by inhibiting specific biological targets. Therefore, using these two isomers (1,3,4-oxadiazole/thiadiazole derivatives) in the pharmaceutical industry could be advantageous because of their chemical and thermal strength.

## 2. Results

### 2.1. Chemistry

**Scheme 1:**
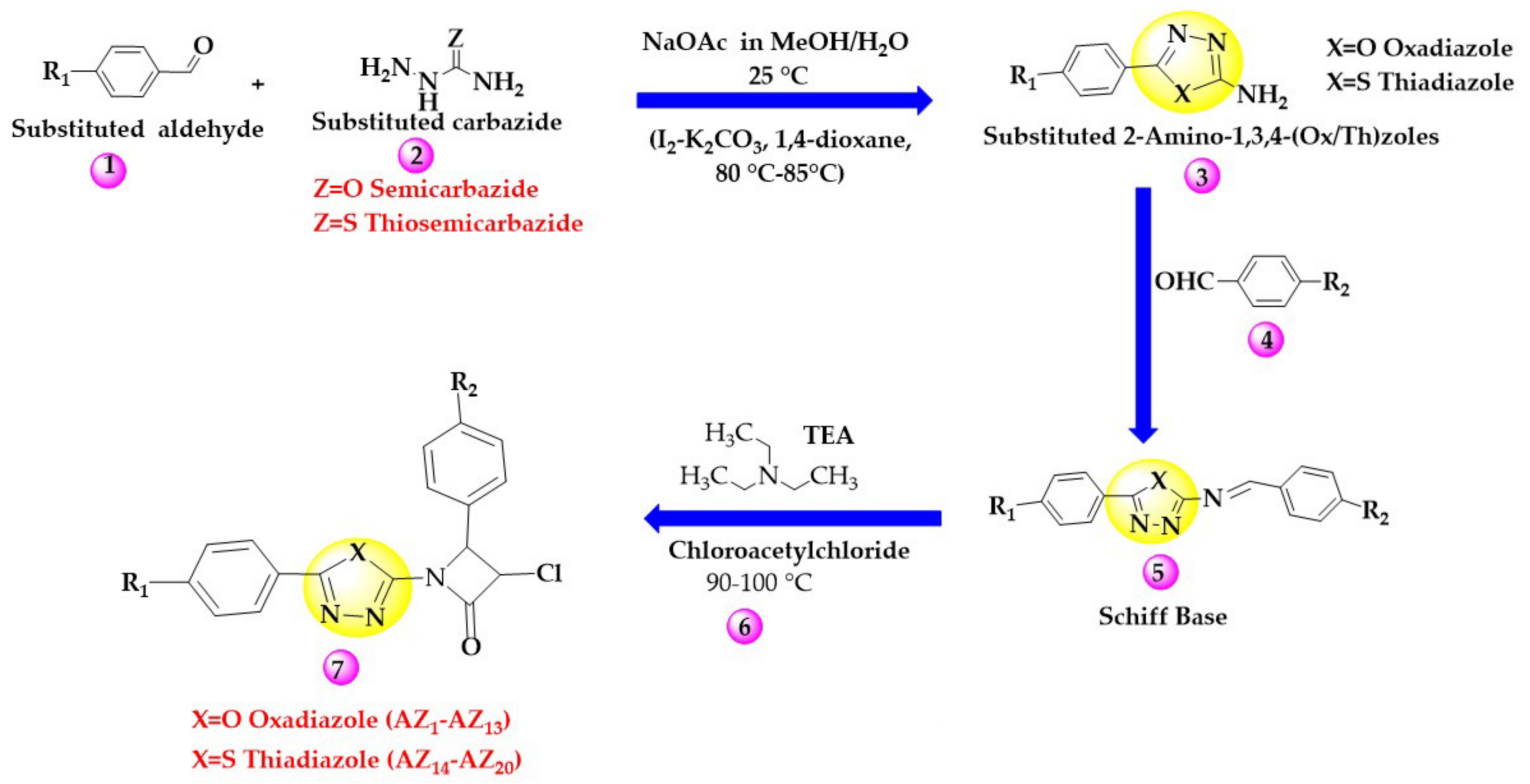
Synthesis of azetidin-2-one derivatives of 1,3,4-oxadiazole/thiadiazole ring (AZ-1–AZ-20).

All synthesized derivatives (AZ-1–AZ-20) were prepared by using a chemical method as shown in (Scheme 1 and Table 1) (substituted azetidin-2-one conjugates). In the first step, 2-amino 1,3,4 oxadiazole/thiadiazole conjugates were synthesized by mixing semi/thio carbazide and sodium acetate with water and stirring well, followed by adding aldehydes in methanol at room temperature. Acetate (glacial) was used as the catalyst to produce Schiff’s bases (intermediates) by treating appropriate aldehydes with 2-amino 1,3,4 oxadiazole/thiadiazole(s). Using the mixture of triethylamine (dropwise) and chloroacetylchloride with vigorous stirring, 4-substitutedphenyl-1,3,4-oxadiazol/Thiadiazol-2-yl)-4-(4-substituted phenyl) azetidin-2-one derivatives were prepared. Synthesized derivatives are summarized in terms of their physicochemical properties and spectral analysis. A variety of spectral techniques, i.e., FT-IR (KBr, cm^−1^), ^1^H-NMR (DMSO-d6, 400 MHz, δ ppm), mass spectra, and elemental analysis, were used to confirm the structures of synthetic analogues (AZ-1–AZ-20). The stretching bands identified at 3020–2824 cm^−1^, 3363–3099 cm^−1^, 1770–1650 cm^−1^, and 1550–1485 cm^−1^, in the IR spectrum indicated the confirmation of C-H (aliphatic stretching), C-H (aromatic stretching), C=O, and C=C (aromatic stretching band), respectively, in the synthesized conjugates. The absorption bands around 1660–1580 cm^−1^, 1350–1228 cm^−1^, and 1180–1040 cm^−1^ in the IR spectrum show different stretching vibrations of C=C (methylene), C-N, and C-C, respectively. Similarly, the presence of a bending absorption band at 689–640 cm^−1^ confirms the C-S-C group in the synthesized molecules. Compound AZ-8 has a stretching absorption band around 535.73 cm^−1^ indicating the presence of C-Br. Compounds AZ-1, AZ-3 and AZ-13 showed absorption bands at 1021.22 cm^−1^–1077.07 and 1500.83–1462.55 cm^−1^, indicating the presence of C-O-C and N=O groups in the synthesized derivatives, respectively. Moreover, the ^1^H-NMR spectrum confirmed that the synthesized derivatives contain aromatic protons, based on multiplet signals between 6.88 and 7.88 ppm. The singlet(s) signals between 5.44–5.45 δ ppm showing the presence of -CH of the azetidine ring were validated in synthesized derivatives at 2.85–3.25 δ ppm, respectively. Observation of the singlet (s) at 4.30 δ ppm in compound AZ-14 demonstrated the presence of a proton of the -NH_2_ group. The methoxy group of Ar–OCH_3_ in the compounds AZ-8, AZ-9, and AZ-16 was validated by the presence of singlet(s) in the range of 3.74–3.83 δ ppm.

### 2.2. Antimicrobial Screening

**Figure 3:**
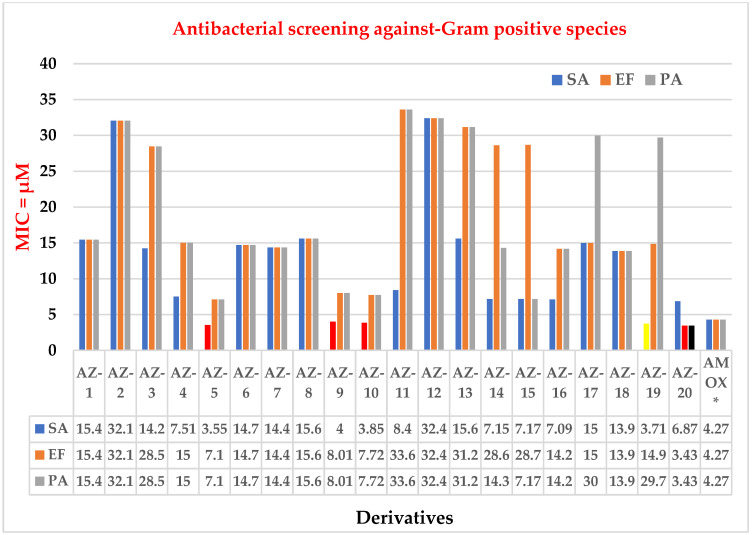
Antibacterial screening against Gram positive species of synthesized derivatives. *-Standard drug.

**Figure 4:**
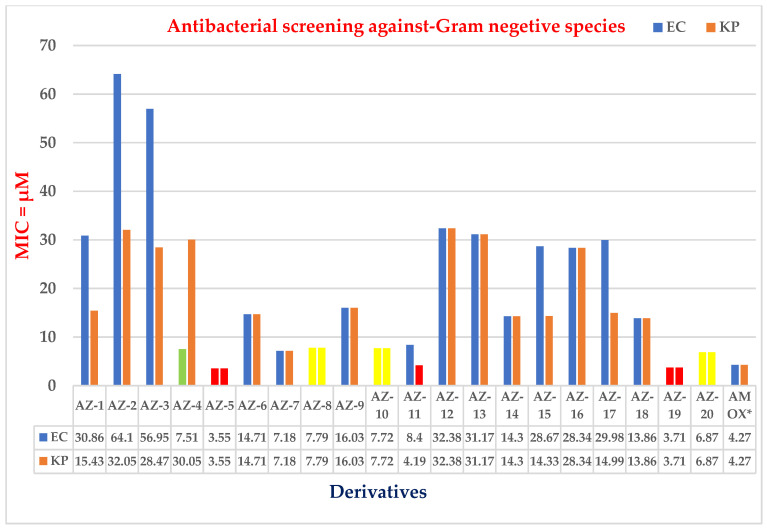
Antibacterial screening against-Gram negative species of synthesized derivatives. *-Standard drug.

**Figure 5:**
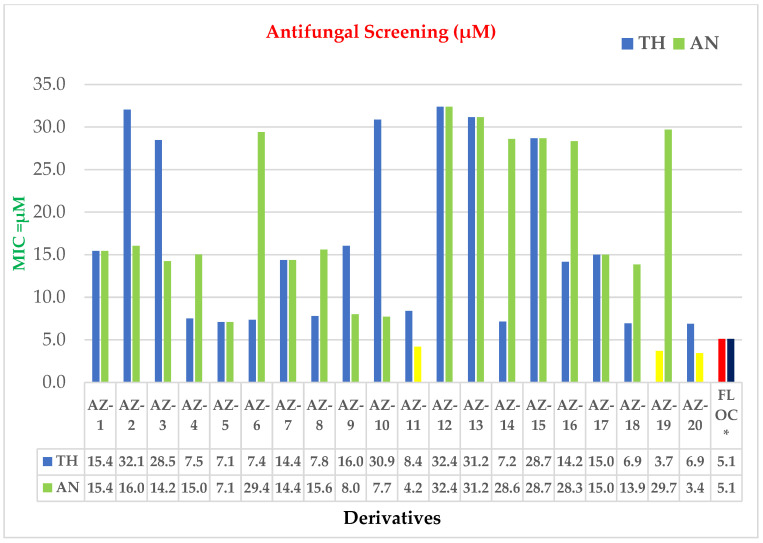
Antifungal screening of (substituted)-1,3,4-oxa/thia-diazol-2-yl)-3-chloro-4-(substituted) azetidin-2-one derivatives. *-Standard drug.

We evaluated the antimicrobial actions of the prepared derivatives using the serial tube dilution method (Table 2, Figure 3, Figure 4 and Figure 5). All derivatives have shown mild to moderate antimicrobial potential against all microorganisms used in in vitro studies. The antibacterial screening outcomes revealed that derivatives AZ-4, 5, 11, 14, 15, and AZ-16 were moderately active against all Gram +ve as well as Gram -ve bacterial strains with MIC values 3.85 µM–7.51 µM, respectively. Further screening evaluates that conjugates AZ-5 and AZ-10 are significantly potent against *S. Aureus* with MICsa = 3.55 µM and 3.85 µM values, respectively. Similarly, another derivative AZ 09 showed its potential against *S. aureus* (MICsa = 4.00 µM), *E. faecalis* (MICef = 8.01 µM), *P. aeruginosa* (MICkp = 8.01 µM), and *A. niger* (MICan = 8.01 µM). Compounds AZ-7 and AZ-8 showed moderate antibacterial potential against Gram-negative (-ve) bacteria (s) *E. coli* and *K. pneumoniae* with their MICec/kp = 7.18 µM and 7.79 µM, respectively. Derivative AZ-19 showed antibacterial as well as antifungal potential against *Escherichia coli*, (MICec = 3.71 µM) *K. pneumoniae* (MICkp = 3.71 µM) and fungal strain *T. Harzianum* (MICth = 3.71 µM), respectively. Overall, compounds AZ 09, 10, and AZ-19 were found to be the most active antibacterial conjugates.

Antibacterial results of the synthesized molecules were comparable to those of the reference drug (amoxicillin), whereas antifungal screening of the synthetic conjugates were evaluated against both strains *T. harzianum* and *A. niger* compared to the standard drug (fluconazole). The antifungal screening outcomes revealed that derivatives AZ-4, 5, and AZ-6, 11, 18, and AZ-20 were moderately active against both fungal strains, i.e., *T. harzianum* and *A. niger* with MIC values 3.43 µM–7.51 µM, respectively. Compound AZ-11 and AZ-20 were remarkably effective against *A. niger* with MIC values 4.19 and 3.42 µM, whereas derivative AZ-19 was found as the most potent antifungal against T. harzianum in comparison to standard drugs. Overall compounds AZ-10, 19, and AZ-20 show promising antimicrobial potential in comparison to the reference drug.

The results from the minimum bactericidal concentration (MBC) test indicate that the synthesized compounds have both bacterial-inhibiting and bacterial-killing effects against the selected bacterial strains. MBC of synthesized azetidin-2-one derivatives of the 1,3,4-oxadiazole/thiadiazole ring was often three times greater than the MIC values, demonstrating that the synthesized compounds were bacteriostatic. (Table 3). All derivatives have shown mild to moderate antibacterial potential against bacterial strains. The MBC screening outcomes revealed that derivatives AZ-5, 9, 11, and AZ-20 were moderately active against all Gram +ve as well Gram -ve bacterial strains with MIC values 4.19 µM–13.74 µM, respectively. Out of all active derivatives, AZ-20 showed remarkable antibacterial potential against Gram +ve as well as Gram -ve bacterial strains. Therefore, these prepared conjugates could be used as lead structures to develop new antimicrobial agents with improved activity. Additionally, they can help reduce the risk of developing drug-resistant bacteria, which can be a major problem in cancer patients.

### 2.3. In-Vitro Cytotoxic Assay (MTT Assay)

MCF-7 cell lines (breast cancer) were used to assess the efficacy of synthesized derivatives (Table 4). Synthesized conjugates (AZ-1–AZ-20) produced a significant amount of apoptosis and cytotoxicity. The preliminary screening of a total of 20 synthesized compounds from the chemical scheme shown as **01** led to only a few derivatives shown cytotoxic potential against the breast cancer cell line (MCF-7). The data obtained from preliminary screening showed highly cytotoxic effects against the MCF-7 cell line with values ranging from 89.84% to 94.76%. Cell viability was measured by MTT assay at different concentrations (0.1 μM, 0.5 μM, 1 μM, 2 μM) and time points. Using synthesized conjugates (AZ-1–AZ-20) as anticancer potential with MCF-7 found that there was a significant increase in cell viability with higher concentrations of synthesized derivatives. In vitro cytotoxicity screening (MTTS assay) revealed that derivatives AZ-5, 9, 10, 14, and AZ-19 demonstrated high efficacy with the percentage of inhibition at different concentration ranges (0.1 μM, 0.5 μM, 1 μM, 2 μM) of 93.28%, 90.56%, 93.14%, 89.84%, and 94.76%, respectively, as compared to doxorubicin (99.98%) as a standard drug, for treating breast cancer. Morphological changes in the cells treated with these agents were assessed by staining with 0.05% crystal violet solution followed by microscopic examination. The anticancer activity measured using MTT assay showed that almost all derivatives exhibited significant anticancer activity as compared to control cells.

### 2.4. In-Vitro Antioxidant Evaluation

**Figure 6:**
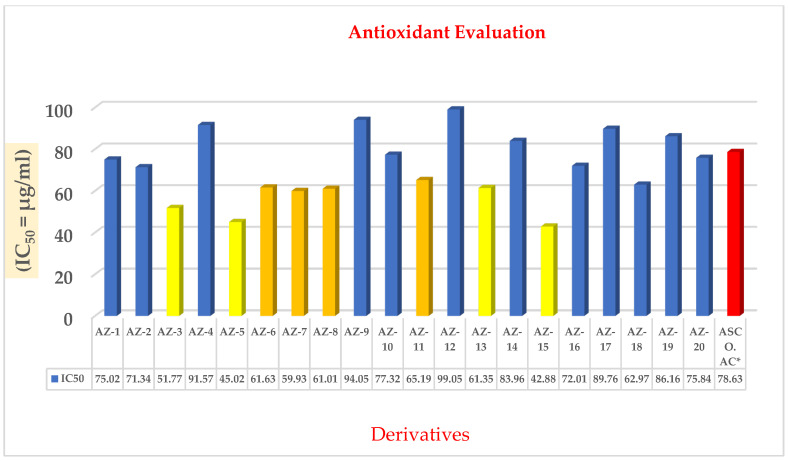
Antioxidant potentials of the synthesized derivatives. *-Standard drug=Ascorbic acid.

The antioxidant potentials of the synthesized conjugates were evaluated by free radical oxidation assay mechanisms (DPPH assay (methanolic) at 517 nm absorption band), respectively. The antioxidant assay is based on the chain-breaking mechanism of DPPH the hydroxyl radical, a natural regulator of the antioxidant defense system. Free radicals such as DPPH can be changed into a diamagnetic state by accepting H^+^ ions or electron radicals from antioxidants. With the formation of a bond between DPPH radicals and antioxidant agents, the color strength of the solution decreases. Increased antioxidant strength increases the DPPH radical’s ability to absorb electrons, decreasing the intensity of the purple solution to colorless, as measured by spectrophotometry at 517 nm. Calculations were made for all synthesized molecules to determine their IC_50_ (μg/mL). The antioxidant screening revealed that most of the synthesized derivatives were more potent than the standard drug, indicating greater stability and effectiveness. As a result of the antioxidant screening, compounds AZ 05 and AZ-15 (IC_50_ = 45.02 μg/mL and 42.88 μg/mL respectively) were found to be the greatest potential. Figure 6 illustrates the results of the antioxidant evaluation.

### 2.5. Structure-Activity Relationship

**Figure 7:**
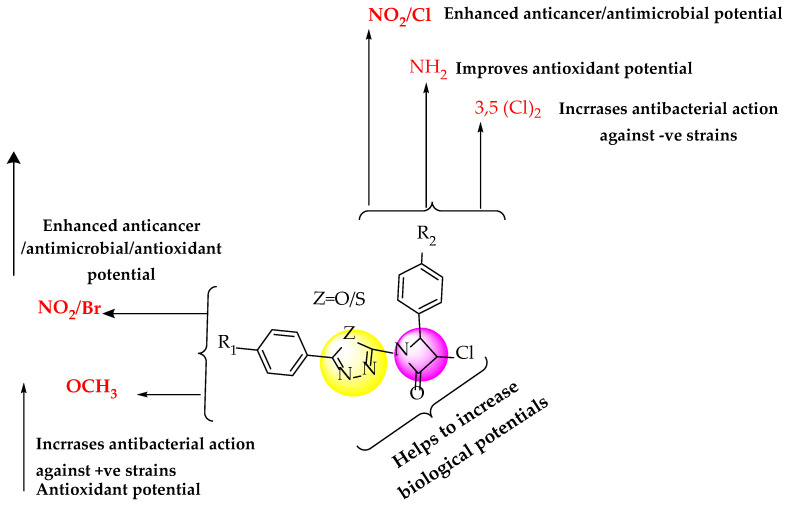
SAR studies of synthesized azetidin-2-one derivatives of 1,3,4-oxadiazole/thiadiazole ring.

Analyzing the structures of synthesized derivatives for anticancer, antimicrobial, and antioxidant activities reveals the following SAR study (Figure 7):

1. The different substitution and presence of pharmacophore ring in the final derivatives of 4-substitutedphenyl-1,3,4-oxadiazol/thiadiazol-2-yl)-4-(4-substituted phenyl) azetidin-2-one derivatives played an extremely crucial role in improving the overall biological potentials.
2. The presence of EWGs (Cl/NO_2_/dichloro) at the para position in the synthesized compound AZ-5, AZ-9, AZ-10, and AZ-19 increases anticancer and antimicrobial potential.
3. The presence of EWGs (Cl/dichloro) at the para position in the synthesized compounds showed significant antibacterial potential against gram (-ve) bacterial strains (AZ-5 and AZ-10).
4. Similarly, the presence of the NH_2_ group at the para position in the synthesized compounds (AZ-15) showed remarkable antioxidant action.
5. EWGs-Cl and Br at the para position in the synthesized compound AZ-19 and AZ-20 increased the antifungal potential against *Aspergillus niger* and *T. harzianum.*
6. The presence of OCH_3_ in the synthesized AZ-8 and AZ-9 resulted in antibacterial activity against *K. pneumoniae*.
7. The overall presence of a Cl and NO_2_ combination at the para position of synthesized conjugates showed remarkable anticancer activity against the MCF-7 cancer cell line and potent antimicrobial action (AZ-19).

Therefore, these potent molecules can serve as lead compounds to create novel antimicrobial and anticytotoxic drugs containing less toxicity and high efficacy.

## 3. Material and Methods

The derivatives were synthesized without purification using commercially available analytical grade chemicals, i.e., (E. Merck (Germany) and S. D. Fine Chem. lmt (India). On a melting point apparatus, open glass capillary measurements were made to determine the melting point (M. Pt). TLC glass plates containing silica gel G were used to monitor each synthetic step using the mobile phases, ethyl acetate: petroleum ether (3:1) *, chloroform: methanol: (7:3) **. TMS was used as an internal standard in the NMR measurement of ^1^H spectra on a Bruker Advance III 400 spectrometer. Mass spectra were obtained using Agilent mass spectrometers. A CHN analyzer was used to analyze the elements.

Synthesis: Step 1: Synthesis of substituted 2 amino 1,3,4, (Oxa/Thia) diazoles (3) [37]

A solution containing Semicarbazide hydrochloride (0.5 mol)/thiosemicarbazides (0.5 mol) (02) was added to water (10 mL) and stirred well before being added to methanol at room temperature. Under reduced pressure, the string was continued until the solvent had not completely evaporated. The residue was then treated with 1,4-dioxane and K_2_CO_3_ (1.5 mol) and iodine (0.5 mol) for 4–6 h at 80–85 °C. A TLC analysis was used to check and monitor the entire reaction step by step. The solution was treated with 5% Na_2_S_2_O_3_ (30 mL) after cooling, and the solution was extracted with CH_2_Cl_2_/MeOH (5:1). Anhydrous sodium sulfate was used to dry and concentrate the combined organic layer. The given residue was purified through a recrystallization process to obtain the corresponding 2 amino 1,3,4, (oxa/thia) diazoles (03) in 80–90% yield.

Step 2: General procedure for the synthesis of Schiff bases (5)

In ethanolic solution, 2-amino-1,3,4, (oxa/thia) diazoles (03) (0.5 mol) were refluxed with different aromatic aldehydes (4) (0.5 mol). To complete the reaction, a small amount of GAA (2–3 mL) (dehydrating agent) was mixed into the reaction solution and the whole solution was refluxed for 7–8 h, or until the reaction was complete. The completion of the reaction was identified by TLC. After the reaction was complete, the excess ethanol as a solvent was distilled off and the residue mixture was stirred for 20 min on ice. After filtrating, washing with ice-cold water, drying, and recrystallizing in ethanol, the precipitates were collected.

Step 3: General procedure for synthesis (Substituted)-1,3,4-Oxa/Thia-diazol-2-yl)-3-chloro-4-(Substituted) azetidin-2-one (7)

An ethanolic solution of Schiff bases (5) (0.5 mol) was diluted with triethylamine 2 mL (dropwise) and then chloroacetylchloride (3–4 mL) (6) was added dropwise with vigorous stirring. Eventually, the mixture was refluxed at 90–100 °C for 4 h or until complete. The completion of the reaction was checked by TLC. The solid obtained was filtered several times. After filtrating, washing with ice-cold water, drying, and recrystallizing in ethanol, the precipitates were collected. All physiochemical and Spectroscopic properties of synthesized derivatives (7) are summarized in Table 5.

### 3.1. Biological Procedure (s): MTT Assay

The MTT assay is a cytotoxic test which measures the metabolic activity of the cells. It is a colorimetric assay and based on the reduction of the yellow tetrazolium salt, i.e., MTT into purple formazan product through active mitochondria. The number of active cells present is directly proportional to the total quantity of MTT cleaved and quantification of the same is done by measuring the absorbance using the colorimeter [38].

The selected compounds to be tested for the anticancer profile were dissolved in DMSO to get a range of different concentrations, keeping the concentration of DMSO at <0.1% in all the compounds. Well-maintained MCF-7 cells of breast cancer were seeded in 96 well plates. To these wells, test samples of different concentrations were added and incubated at 37 °C in a 5% CO_2_ incubator for 96 h. Further to these wells, MTT reagent was added and incubate again for 4 h. Finally, the purple formazan product made by the cells was collected and dissolved in DMSO (100 μL/mL). Absorption was noted using a colorimeter at 550 nm. Then the value of percentage inhibitions was calculated in triplicate absorbance.

The percentage of cell growth inhibition (Equation (1)) was calculated with the following equation:

1. MTT Assay Protocol (Steps involved in cell culture) Cell lines were grown for different types such as MCF-7 in RPMI-1640 medium.

Inhibition (%) = (1 − Ab_Sample_/Ab_control_) × 100 (1)

2. Media were supplemented with 10% FBS.
3. MCF-7 cell lines were cultured and maintained in DMEM medium supplemented with 10% FBS.
4. The culture was kept in a 5% CO_2_ atmosphere with controlled humidity at 37 °C.
5. A stock solution of test conjugates was made in DMSO and added to the cell culture at desired concentrations. The final culture was not diluted more than 1:1000 with DMSO.

### 3.2. In Vitro Antimicrobial Assay

Synthesized conjugates were tested for antimicrobial activity using fluconazole and amoxicillin as standard antibiotics using the serial tube dilution method [39]. This study used Gram +ve (MTCC-3160 (*S. aureus*), Gram +ve (MTCC-441 (*E. faecalis*), MTCC-3541 (*P. aeruginosa)* and Gram -ve (MTCC-443 (*E. coli*), and Gram -ve (MTCC-9024 (*K. pneumoniae*)] bacteria. In this study, MTCC-3683 (*T. harzianum*) and MTCC-281 (*A. niger*) strains were tested for their antifungal potential. The double-strength nutrient broth I.P. (for bacteria) or sabouraud dextrose broth I.P. (for fungi) were used to test their antimicrobial activity [40]. A dimethyl sulfoxide stock solution was prepared for the test and standard drugs. Additionally, dimethyl sulfoxide was added to the test medium as a control set at the same dilutions. Results were recorded in MIC after incubating the samples at 25 ± 1 °C (7 days) for *A. niger*, at 25 ± 1 °C (36 h) for *T. harzianum* and at 37 ± 1 °C (24 h) for bacteria (s), respectively. The MIC for the tested compound was observed as the lowest conc. of the derivative that prevented microorganism growth inside the tube. The antimicrobial potential was calculated in micromolar [µM] for all the synthesized compounds by using the following equation (Equation (2)).

Micromolar [µM] = MIC/MW × 1000(2)

#### Minimum Bactericidal Concentration (MBC)

In total, 100 µL of culture was sub-cultured to a new medium from the tubes that had no changes in the MIC determination in order to calculate the minimum bactericidal concentration. Lowest concentrations of compounds that result in a 99.9% end point reduction were represented by MBC values. (Table 5) The minimum bactericidal concentration was calculated in micromolar [µM] for all the synthesized compounds by using the following equation (Equation (2)) [41].

### 3.3. In Vitro Antioxidant Assay

All synthesized derivatives (07) were evaluated for their antioxidant properties using the DPPH free radical scavenging model [42]. We prepared diluted solutions of the synthesized conjugates in methanol at 25 μg/mL, 50 μg/mL, 75 μg/mL, and 100 μg/mL, individually, and added equal quantities of methanolic DPPH solution (0.0039%). A UV-visible double-beam spectrophotometer measuring 517 nm absorbance was used to measure the above solution’s absorbance after 30 min in the dark. Data presented here represent the mean IC_50_ based on the average of at least three observations.

## 4. Conclusions

The goal of this research was to develop new anticancer, antimicrobial, and antioxidant drugs by using azetidin-2-one derivatives of the 1,3,4-oxadiazole/thiadiazole ring. Because of their versatile biological action, the goal of this research has been to integrate the azetidin-2-one moiety into the oxadiazole ring to achieve 1-(5-(4-substituted phenyl)-1,3,4-oxadiazol/thiadiazol-2-yl)-4-(4-substituted phenyl) for the exploration of altered biological interactions. In vitro cytotoxicity screening (MTTS assay) revealed that derivatives AZ-5, 9, 10, 14, and AZ-19 demonstrated high efficacy with the percentage of inhibition at different concentration ranges (0.1 μM, 0.5 μM, 1 μM, 2 μM) of 89% to 94% μM as compared to doxorubicin as the standard drug. The antimicrobial evaluation showed that compounds AZ-10, 19, and AZ-20 were found to have significant antimicrobial potential with MIC ranges of 3.34 µM to 3.71 µM in comparison to reference drugs having 4.29 µM to 5.10 µM. Based on antioxidant screening, most of the synthetic derivatives showed greater stability and effectiveness than the standard drug. According to the antioxidant screening, compounds AZ 05 and AZ-15 (IC_50_ = 45.02 μg/mL and 42.88 μg/mL, respectively) showed the greatest potency, as compared to ascorbic acid (IC_50_ = 78.63 g/mL). SAR studies of a synthesized novel series revealed that para-substituted halogen and nitro derivatives have remarkable potential against MCF-7 cancer cell lines and different microbial strains.

## Figures and Tables

**Table 1:** Azetidin-2-one derivatives of 1,3,4-oxadiazole/thiadiazole ring.

COMPOUNDs	R_1_	R_2_
AZ-1	Cl	NO_2_
AZ-2	Cl	OCH_3_
AZ-3	Cl	Br
AZ-4	NO_2_	NO_2_
AZ-5	NO_2_	3,5 (Cl)_2_
AZ-6	OCH_3_	3,5 (Cl)_2_
AZ-7	OCH_3_	Br
AZ-8	OCH_3_	NO_2_
AZ-9	OCH_3_	Cl
AZ-10	NO_2_	Cl
AZ-11	OCH_3_	OH
AZ-12	OCH_3_	OCH_3_
AZ-13	NO_2_	OCH_3_
AZ-14	Br	OH
AZ-15	Br	NH_2_
AZ-16	OCH_3_	3,5 (Cl)_2_
AZ-17	OCH_3_	NO_2_
AZ-18	OCH_3_	Br
AZ-19	Cl	NO_2_
AZ-20	Br	Cl

**Table 2:** Antimicrobial screening (µM) of (substituted)-1,3,4-oxa/thia-diazol-2-yl)-3-chloro-4-(substituted) azetidin-2-one derivatives.

Antimicrobial Screening							
Comp.	Gram-Positive Bacteria(s)			Gram Negative Bacteria(s)		Fungal Strains	
	SA	EF	PA	EC	KP	TH	AN
AZ-1	15.43	15.43	15.43	30.86	15.43	15.43	15.43
AZ-2	32.05	32.05	32.05	64.10	32.05	32.05	16.03
AZ-3	14.24	28.47	28.47	56.95	28.47	28.47	14.24
AZ-4	7.51	15.02	15.02	7.51	30.05	7.51	15.02
AZ-5	3.55	7.10	7.10	3.55	3.55	7.10	7.10
AZ-6	14.71	14.71	14.71	14.71	14.71	7.35	29.41
AZ-7	14.37	14.37	14.37	7.18	7.18	14.37	14.37
AZ-8	15.59	15.59	15.59	7.79	7.79	7.79	15.59
AZ-9	4.00	8.01	8.01	16.03	16.03	16.03	8.01
AZ-10	3.85	7.72	7.72	7.72	7.72	30.86	7.72
AZ-11	8.40	33.60	33.60	8.40	4.19	8.40	4.19
AZ-12	32.38	32.38	32.38	32.38	32.38	32.38	32.38
AZ-13	15.59	31.17	31.17	31.17	31.17	31.17	31.17
AZ-14	7.15	28.60	14.30	14.30	14.30	7.15	28.60
AZ-15	7.17	28.67	7.17	28.67	14.33	28.67	28.67
AZ-16	7.09	14.17	14.17	28.34	28.34	14.17	28.34
AZ-17	14.99	14.99	29.98	29.98	14.99	14.99	14.99
AZ-18	13.86	13.86	13.86	13.86	13.86	6.93	13.86
AZ-19	3.71	14.85	29.69	3.71	3.71	3.71	29.69
AZ-20	6.87	3.43	3.43	6.87	6.87	6.87	3.43
Std Drug	4.29 *	4.29 *	4.29 *	4.29 *	4.29 *	5.10 **	5.10 **

PA: *Pseudomonas aeruginosa* SA: *Staphylococcus aureus* KP: *Klebsiella pneumoniae* EF: *Enterococcus faecalis* EC: *Escherichia coli* TH: *Trichoderma harzianum* AN: *Aspergillus niger*. Std Drug-*Amoxicillin* *, Flu*conazole* **.

**Table 3:** Minimum bactericidal concentration (µM) screening of azetidin-2-one derivatives of 1,3,4-oxadiazole/thiadiazole ring.

Minimum Bactericidal Concentration (µM) Evaluation					
Comp.	Gram Positive Bacteria (s)			Gram Negative Bacteria (s)	
	SA	EF	PA	EC	KP
AZ-1	30.86	30.86	30.86	30.86	30.86
AZ-2	64.10	64.10	64.10	64.10	64.10
AZ-3	28.47	56.95	56.95	56.95	56.95
AZ-4	15.02	30.05	30.05	15.02	30.05
AZ-5	7.10	7.10	7.10	14.40	14.20
AZ-6	29.42	14.71	29.42	29.42	29.42
AZ-7	14.37	14.37	14.37	14.37	14.37
AZ-8	15.59	15.59	15.59	31.18	31.18
AZ-9	8.01	16.03	16.03	16.03	16.03
AZ-10	7.72	14.30	14.30	28.67	28.67
AZ-11	8.40	33.60	33.60	16.80	8.40
AZ-12	32.38	32.38	32.38	32.38	32.38
AZ-13	15.59	31.17	62.34	62.34	31.17
AZ-14	14.30	28.60	14.30	14.30	14.30
AZ-15	14.33	28.67	14.33	43.10	14.33
AZ-16	14.17	28.34	28.34	28.34	28.34
AZ-17	14.99	14.99	29.98	29.98	14.99
AZ-18	13.86	13.86	13.86	13.86	13.86
AZ-19	14.85	14.85	29.69	14.85	14.85
AZ-20	6.87	6.87	6.87	13.74	13.74
Amox.	4.27	4.27	4.27	4.27	4.27

**Table 4:** In vitro cytotoxicity screening against MCF-7 of (substituted)-1,3,4-oxa/thia-diazol-2-yl)-3-chloro-4-(substituted) azetidin-2-one derivatives.

Comp.	Conc.	Absorbance	% of Inhibition	IC_50_	Comp.	Conc.	Absorbance	% of Inhibition	IC_50_
AZ-1	0	1.74 ± 0.02		4.89	AZ-11	0	2.44 ± 0.02		2.25
	0.1	1.41 ± 0.03	18.77			0.1	1.76 ± 0.02	27.84	
	0.5	1.31 ± 0.04	24.57			0.5	1.14 ± 0.04	53.32	
	1	1.21 ± 0.05	30.40			1	0.92 ± 0.03	62.09	
	2	0.95 ± 0.03	45.08			2	0.72 ± 0.07	70.36	
AZ-2	0	1.67 ± 0.02		2.16	AZ-12	0	1.91 ± 0.02		2.55
	0.1	1.26 ± 0.04	24.65			0.1	1.44 ± 0.04	24.52	
	0.5	0.94 ± 0.03	43.78			0.5	1.22 ± 0.01	35.98	
	1	0.41 ± 0.03	75.43			1	0.82 ± 0.02	57.06	
	2	0.26 ± 0.02	84.55			2	0.42 ± 0.05	78.29	
AZ-3	0	1.66 ± 0.02		3.72	AZ-13	0	2.65 ± 0.02		1.76
	0.1	1.55 ± 0.04	6.69			0.1	1.87 ± 0.03	29.29	
	0.5	1.11 ± 0.02	33.20			0.5	1.01 ± 0.02	61.79	
	1	0.88 ± 0.02	46.84			1	0.59 ± 0.06	77.83	
	2	0.88 ± 0.01	47.23			2	0.40 ± 0.05	84.87	
AZ-4	0	1.73 ± 0.02		4.49	AZ-14	0	3.40 ± 0.03		1.96
	0.1	1.40 ± 0.02	19.31			0.1	2.27 ± 0.01	33.27	
	0.5	1.22 ± 0.01	29.64			0.5	1.74 ± 0.01	48.68	
	1	1.09 ± 0.02	37.07			1	1.05 ± 0.01	69.06	
	2	0.95 ± 0.05	45.43			2	0.35 ± 0.01	89.85	
AZ-5	0	1.73 ± 0.05		2.01	AZ-15	0	2.83 ± 0.02		1.93
	0.1	1.49 ± 0.03	14.18			0.1	1.87 ± 0.02	34.13	
	0.5	0.66 ± 0.04	61.66			0.5	1.22 ± 0.01	57.00	
	1	0.31 ± 0.04	82.32			1	1.05 ± 0.02	63.02	
	2	0.12 ± 0.07	93.29			2	0.70 ± 0.02	75.20	
AZ-6	0	2.30 ± 0.04		2.04	AZ-16	0	2.07 ± 0.02		2.22
	0.1	1.56 ± 0.04	32.29			0.1	1.63 ± 0.01	21.50	
	0.5	1.09 ± 0.03	52.59			0.5	1.05 ± 0.01	49.29	
	1	0.90 ± 0.02	60.80			1	0.68 ± 0.02	67.35	
	2	0.37 ± 0.03	84.12			2	0.32 ± 0.02	84.55	
AZ-7	0	1.89 ± 0.04		2.70	AZ-17	0	1.72 ± 0.02		2.91
	0.1	1.27 ± 0.04	33.05			0.1	1.41 ± 0.01	18.45	
	0.5	1.08 ± 0.03	42.97			0.5	1.10 ± 0.01	36.49	
	1	0.89 ± 0.03	53.04			1	0.78 ± 0.01	54.50	
	2	0.70 ± 0.06	62.85			2	0.61 ± 0.02	64.67	
AZ-8	0	2.65 ± 0.02		1.76	AZ-18	0	1.78 ± 0.02		2.89
	0.1	1.87 ± 0.03	29.29			0.1	1.49 ± 0.02	16.13	
	0.5	1.01 ± 0.02	61.79			0.5	1.09 ± 0.01	38.57	
	1	0.59 ± 0.07	77.83			1	0.91 ± 0.02	48.53	
	2	0.40 ± 0.04	84.87			2	0.54 ± 0.02	69.63	
AZ-9	0	2.84 ± 0.03		1.62	AZ-19	0	2.29 ± 0.02		2.70
	0.1	1.80 ± 0.03	36.80			0.1	1.60 ± 0.02	30.18	
	0.5	1.09 ± 0.03	61.50			0.5	1.17 ± 0.02	48.84	
	1	0.82 ± 0.05	70.99			1	0.96 ± 0.03	58.12	
	2	0.27 ± 0.05	90.56			2	0.12 ± 0.04	94.76	
AZ-10	0	2.89 ± 0.04		1.03	AZ-20	0	2.28 ± 0.02		2.71
	0.1	1.40 ± 0.02	51.46			0.1	1.81 ± 0.01	20.44	
	0.5	1.07 ± 0.02	62.87			0.5	1.64 ± 0.03	28.05	
	1	0.88 ± 0.03	69.64			1	1.02 ± 0.02	55.27	
	2	0.20 ± 0.08	93.15			2	0.48 ± 0.03	79.11	
Dox. (Ref. Drug)	0	3.28 ± 0.02		0.05					
	0.1	1.73 ± 0.01	48.88						
	0.5	1.03 ± 0.02	65.58						
	1	0.76 ± 0.02	83.12						
	2	0.09 ± 0.03	98.76						

**Table 5:** Spectroscopic properties of synthesized derivatives.

3-chloro-1-(5-(4-chlorophenyl)-1,3,4-oxadiazol-2-yl)-4-(4-methoxyphenyl)azetidin-2-one. Mol. Formula C_18_H_13_Cl_2_N_3_O_3_, Mol.Wt: 390, Color Dark Yellowish, % Yield 59%, M. Pt 243–245 °C, Rf Value 0.7 *. IR (KBr pellets) cm^−1^: (C-H stretching, aromatic), 3077.30, (C=O stretching, azetidine ring), 1730.62 (C=N stretching, imine group), 1644.93, (C=C stretching, aromatic ring), 1526.07, (C-O-C stretching, Oxadiazole ring), 1022.24, (C=C stretching, methylene group), 1596.85, (N=O stretching, Nitro Group), 1461.89, (C-Cl stretching, azetidine ring). 710.86, (C-Cl stretching, benzene ring). 811.7. ^1^H NMR (DMSO-d6, 400 MHz, δ ppm) δ 7.50–7.46 (m, 2H), 7.44–7.40 (m, 2H), 7.28–7.24 (m, 2H), 6.93–6.89 (m, 2H), 5.97 (s, 1H), 5.44 (s, 1H), 3.83–3.79 (m, 3H). CHN (Elemental analysis) calculated: C, 55.40; H, 3.36; N, 10.77. Found: C, 56.01; H, 3.06; N, 09.17.
4-(4-bromophenyl)-3-chloro-1-(5-(4-chlorophenyl)-1,3,4-oxadiazol-2-yl)azetidin-2-one. Mol. Formula C_17_H_10_BrCl_2_N_3_O_2_, Mol.Wt: 439, Color Reddish, % Yield 47%, M. Pt 213–215 °C, Rf Value 0.5 *. IR (KBr pellets) cm^−1^: (C-H stretching, aromatic), 3104.92, (C=O stretching, azetidine ring), 1719.27 (C=N stretching, imine group), 1640.54, (C=C stretching, aromatic ring), 1566.02, (C-O-C stretching, Oxadiazole ring), 1077.07, (C=C stretching, methylene group), 1623.75, (N=O stretching, Nitro Group), 1482.73, (C-N stretching, Oxadiazole ring), 1623.75, (C-Cl stretching, azetidine ring). 759.76, (C-Cl stretching, benzene ring). 591.54. ^1^H NMR (DMSO-d6, 400 MHz, δ ppm) δ 7.50–7.46 (m, 4H), 7.44–7.40 (m, 2H), 7.20–7.16 (m, 2H), 6.03 (s, 1H), 5.44 (s, 1H). CHN (Elemental analysis) calculated: C, 46.50; H, 2.30; N, 9.57. Found: C, 45.78; H, 2.10; N, 9.37.
3-chloro-4-(4-nitrophenyl)-1-(5-(4-nitrophenyl)-1,3,4-oxadiazol-2-yl)azetidin-2-one, Mol. Formula C_17_H_10_ClN_5_O_6_, Mol.Wt: 416, Color Light green, % Yield 61%, M. Pt 260–262 °C, Rf Value 0.7 * (IR (KBr pellets) cm^−1^: (C-H stretching, aromatic), 3094.57, (C=O stretching, azetidine ring), 1719.01 (C=N stretching, imine group), 1624.49, (C=C stretching, aromatic ring), 1513.82, (C-O-C stretching, Oxadiazole ring), 994.19, (C=C stretching, methylene group), 1513.82, (N=O stretching, Nitro Group), 1478.35, (C-Cl stretching, azetidine ring). 817.24 cm^−1^. ^1^H NMR (DMSO-d6, 400 MHz, δ ppm) δ 8.34–8.30 (m, 2H), 8.22–8.18 (m, 2H), 7.88–7.84 (m, 2H), 7.59–7.55 (m, 2H), 5.99 (s, 1H), 5.44 (s, 1H).). CHN (Elemental analysis) calculated: C, 49.11; H, 2.42; N, 16.85. Found: C, 48.91; H, 2.12; N, 16.05.
3-chloro-4-(3,5-dichlorophenyl)-1-(5-(4-nitrophenyl)-1,3,4-oxadiazol-2-yl)azetidin-2-one. Mol. Formula C_17_H_9_Cl_3_N_4_O_4_, Mol.Wt: 440, Color Yellowish, % Yield 78%, M. Pt 216–218 °C, Rf Value 0.8 *. (IR (KBr pellets) cm^−1^: (C-H stretching, aromatic), 3077.02, (C=O stretching, azetidine ring), 1717.83 (C=N stretching, imine group), 1638.32, (C=C stretching, aromatic ring), 1541.00, (C-O-C stretching, Oxadiazole ring), 994.83, (C=C stretching, methylene group), 1514.92, (N=O stretching, Nitro Group), 1478.35, (C-Cl stretching, azetidine ring). 711.63, (C-Cl stretching, Benzene ring). 581.02. ^1^H NMR (DMSO-d6, 400 MHz, δ ppm) δ 8.34–8.30 (m, 2H), 7.88–7.84 (m, 2H), 7.33–7.26 (m, 3H), 5.92 (s, 1H), 5.44 (s, 1H).). CHN (Elemental analysis) calculated: C, 46.44; H, 2.06; N, 12.74. Found: C, 46.04; H, 2.26; N, 12.34.
3-chloro-4-(3,5-dichlorophenyl)-1-(5-(4-methoxyphenyl)-1,3,4-oxadiazol-2-yl)azetidin-2-one. Mol. Formula C_18_H_12_Cl_3_N_3_O_3_, Mol.Wt: 425, Color White, % Yield 56%, M. Pt 273–275 °C Rf Value 0.7 *. IR (KBr pellets) cm^−1^: (C-H stretching, aromatic), 3080.90, (C=O stretching, azetidine ring), 1696.15 (C=N stretching, imine group), 1642.73, (C=C stretching, aromatic ring), 1604.64, (C-O-C stretching, Oxadiazole ring), 991.01, (C-N stretching, imine group), 1241.96, (N=O stretching, Nitro Group), 1511.02, (C-Cl stretching, azetidine ring). 609.30, (C-Cl stretching, benzene ring). 824.03. ^1^H NMR (DMSO-d6, 400 MHz, δ ppm) δ 7.60–7.56 (m, 2H), 7.33–7.26 (m, 3H), 7.06–7.02 (m, 2H), 5.93 (s, 1H), 5.44 (s, 1H), 3.83–3.79 (m, 3H). CHN (Elemental analysis) calculated: C, 50.91; H, 2.85; N, 9.90. Found: C,49.89; H, 2.45; N, 9.67.
4-(4-bromophenyl)-3-chloro-1-(5-(4-methoxyphenyl)-1,3,4-oxadiazol-2-yl)azetidin-2-one, Mol. Formula C_18_H_13_BrClN_3_O_3_, Mol.Wt: 435, Color Reddish, % Yield 70%, M. Pt 256–258 °C, Rf Value 0.8 **. IR (KBr pellets) cm^−1^: (C-H stretching, aromatic), 3090.44, (C=O stretching, azetidine ring), 1728.20 (C=N stretching, imine group), 1628.34, (C=C stretching, aromatic ring), 1600.08, (C-O-C stretching, Oxadiazole ring), 993.30, (C=C stretching, methylene group), 1646.09, (C-N stretching, imine group), 1230.74, (N=O stretching, Nitro Group), 1525.68 (C-N stretching, Oxadiazole ring), 1160.29, (C-Cl stretching, azetidine ring). 833.21, (C-Br stretching, benzene ring). 535.73. ^1^H NMR (DMSO-d6, 400 MHz, δ ppm) δ 7.60–7.56 (m, 2H), 7.49–7.45 (m, 2H), 7.22–7.18 (m, 2H), 7.06–7.02 (m, 2H), 5.89 (s, 1H), 5.44 (s, 1H), 3.83–3.79 (m, 3H).). CHN (Elemental analysis) calculated: C, 49.74; H, 3.01; N, 9.67. Found: C, 49.14; H, 2.71; N, 9.27.
3-chloro-1-(5-(4-methoxyphenyl)-1,3,4-oxadiazol-2-yl)-4-(4-nitrophenyl)azetidin-2-one, Mol. Formula C_18_H_13_ClN_4_O_5_, Mol.Wt: 401, Color Cream, % Yield 49%, M. Pt 210–203 °C, Rf Value 0.7 *. IR (KBr pellets) cm^−1^: (C-H stretching, aromatic), 3094.12, (C=O stretching, azetidine ring), 1724.33, C=N stretching, imine group), 1627.23, (C=C stretching, aromatic ring), 1519.86, (C-O-C stretching, Oxadiazole ring), 1466.14, (C=C stretching, methylene group), 1590.50, (C-N stretching, imine group), 1083.45, (N=O stretching, Nitro Group), 1519.86, (C-Cl stretching, azetidine ring). 831.78, (C-Cl stretching, benzene ring). 715 ^1^H NMR (DMSO-d6, 400 MHz, δ ppm) δ 8.22–8.18 (m, 2H), 7.60–7.55 (m, 4H), 7.06–7.02 (m, 2H), 6.00 (s, 1H), 5.44 (s, 1H), 3.83–3.79 (m, 3H). CHN (Elemental analysis) calculated: C, 53.94; H, 3.27; N, 13.98. Found: C, 53.14; H, 3.07; N, 13.38.
3-chloro-4-(4-chlorophenyl)-1-(5-(4-methoxyphenyl)-1,3,4-oxadiazol-2-yl)azetidin-2-one. Mol. Formula C_18_H_13_Cl_2_N_3_O_3_, Mol.Wt: 390, Color Brownish, % Yield 54%, M. Pt 226–228 °C, Rf Value 0.5 **. IR (KBr pellets) cm^−1^: (C-H stretching, aromatic), 3080.56, (C=O stretching, azetidine ring), 1696.14 (C=N stretching, imine group), 1651.18, (C=C stretching, aromatic ring), 1603.86, (C-O-C stretching, Oxadiazole ring), 1019.52, (C-N stretching, imine group), 1163.73, (N=O stretching, Nitro Group), 1511.19, (C-N stretching, Oxadiazole ring), 1242.50, (C-Cl stretching, azetidine ring). 824.64, (C-Cl stretching, benzene ring). 610.60. ^1^H NMR (DMSO-d6, 400 MHz, δ ppm) δ 7.60–7.56 (m, 2H), 7.33–7.29 (m, 2H), 7.27–7.23 (m, 2H), 7.06–7.02 (m, 2H), 5.89 (s, 1H), 5.44 (s, 1H), 3.83–3.79 (m, 3H). CHN (Elemental analysis) calculated: C, 55.40; H, 3.36; N, 10.77. Found: C, 54.99; H, 3.06; N, 10.17.
3-chloro-4-(4-chlorophenyl)-1-(5-(4-nitrophenyl)-1,3,4-oxadiazol-2-yl)azetidin-2-one,. Mol. Formula C_17_H_10_Cl_2_N_4_O_4_, Mol.Wt: 405, Color Cream, % Yield 78%, M. Pt 267–269 °C, Rf Value 0.6 *. IR (KBr pellets) cm^−1^: (C-H stretching, aromatic), 3088.79, (C=O stretching, azetidine ring), 1727.68 (C=N stretching, imine group), 1646.65, (C=C stretching, aromatic ring), 1628.03, (C-O-C stretching, Oxadiazole ring), 1011.50, (C=C stretching, methylene group), 1598.06, (C-N stretching, imine group), 1230.57, (N=O stretching, Nitro Group), 1525.01, (C-Cl stretching, azetidine ring). 832.40. ^1^H NMR (DMSO-d6, 400 MHz, δ ppm) δ 8.34–8.30 (m, 2H), 7.88–7.84 (m, 2H), 7.33–7.29 (m, 2H), 7.27–7.23 (m, 2H), 5.88 (s, 1H), 5.44 (s, 1H).). CHN (Elemental analysis) calculated: C, 50.39; H, 2.49; N, 13.83. Found: C, 50.11; H, 2.07; N, 13.22.
3-chloro-4-(4-hydroxyphenyl)-1-(5-(4-methoxyphenyl)-1,3,4-oxadiazol-2-yl)azetidin-2-one. Mol. Formula C_18_H_14_ClN_3_O_4_, Mol.Wt: 372. Color Cream, % Yield 67%, M. Pt 213–215 °C, Rf Value 0.6 **. IR (KBr pellets) cm^−1^: (C-H stretching, aromatic), 3047.54, (C-OH stretching, benzene ring). 3369.92 (C=O stretching, azetidine ring), 1708.16, (C=N stretching, imine group), 1672.71, (C=C stretching, aromatic ring), 1543.59, (C-O-C stretching, Oxadiazole ring), 1078.82, (C-N stretching, imine group), 1153.83, (C-N stretching, Oxadiazole ring), 1272.32, (C-Cl stretching, azetidine ring). 616.31. ^1^H NMR (DMSO-d6, 400 MHz, δ ppm) δ 7.60–7.56 (m, 2H), 7.16–7.12 (m, 2H), 7.06–7.02 (m, 2H), 6.80–6.76 (m, 2H), 5.82 (s, 1H), 5.44 (s, 1H), 3.83–3.79 (m, 3H), 3.68 (s, 1H). CHN (Elemental analysis) calculated: C, 58.15; H, 3.80; N, 11.30. Found: C, 57.73; H, 3.43; N, 11.01.
3-chloro-4-(4-methoxyphenyl)-1-(5-(4-methoxyphenyl)-1,3,4-oxadiazol-2-yl)azetidin-2-one, Mol. Formula C_19_H_16_ClN_3_O_4_ Mol.Wt: 386. Color White, % Yield 61%, M. Pt 243–245 °C, Rf Value0.6 *. IR (KBr pellets) cm^−1^: (C-H stretching, aromatic), 3120.65, (C=O stretching, azetidine ring), 1727.66 (C=N stretching, imine group), 1644.26, (C=C stretching, aromatic ring), 1610.28, (C-O-C stretching, Oxadiazole ring), 1020.66, (C-N stretching, imine group), 1120.32, (N=O stretching, Nitro Group), 1542.66, (C-Cl stretching, azetidine ring). 767.53. ^1^H NMR (DMSO-d6, 400 MHz, δ ppm) δ 7.60–7.56 (m, 2H), 7.30–7.26 (m, 2H), 7.06–7.02 (m, 2H), 6.92–6.88 (m, 2H), 5.82 (s, 1H), 5.44 (s, 1H), 3.83–3.79 (m, 6H). CHN (Elemental analysis) calculated: C, 59.15; H, 4.18; N, 10.89. Found: C, 58.92; H, 4.02; N, 10.11.
3-chloro-4-(4-methoxyphenyl)-1-(5-(4-nitrophenyl)-1,3,4-oxadiazol-2-yl)azetidin-2-one. Mol. Formula C_18_H_13_ClN_4_O_5_, Mol.Wt: 401, Color Brown, % Yield 59%, M. Pt 219–221 °C, Rf Value 0.7 *. IR (KBr pellets) cm^−1^: (C-H stretching, aromatic), 3056, (C=O stretching, azetidine ring), 1717.65 (C=N stretching, imine group), 1638.15, (C=C stretching, aromatic ring), 1598.12, (C-O-C stretching, Oxadiazole ring), 1092.55, (C-N stretching, imine group), 1152.79, (N=O stretching, Nitro Group), 1500.83, (C-N stretching, Oxadiazole ring), 1179.03, (C-Cl stretching, azetidine ring). 753.62. ^1^H NMR (DMSO-d6, 400 MHz, δ ppm) δ 8.34–8.30 (m, 2H), 7.88–7.84 (m, 2H), 7.30–7.26 (m, 2H), 6.92–6.88 (m, 2H), 5.81 (s, 1H), 5.44 (s, 1H), 3.83–3.79 (m, 3H). CHN (Elemental analysis) calculated: C, 53.94; H, 3.27; N, 13.98. Found: C, 53.12; H, 3.32; N, 13.16.
1-(5-(4-bromophenyl)-1,3,4-thiadiazol-2-yl)-3-chloro-4-(4-hydroxyphenyl)azetidin-2-one, Mol. Formula C_17_H_11_BrClN_3_O_2_S, Mol.Wt:: 437, Color Brown, % Yield 56%, M. Pt 278–280 °C, Rf Value 0.6 *. IR (KBr pellets) cm^−1^: (C-OH stretching, benzene ring). 3363.10 cm^−1^ (C=O stretching, azetidine ring), 1746.76 (C=N stretching, imine group), 1698.51, (C=C stretching, aromatic ring), 1675.56, (C-S-C stretching, Thiadiazole ring), 688.12, (C=C stretching, methylene group), 1617.24, (C-N stretching, imine group), 1205.08, (N=O stretching, Nitro Group), (C-N stretching, Oxadiazole ring), 1374.44 cm^−1^, (C-Cl stretching, azetidine ring). 682.89, (C-Cl stretching, benzene ring) 715. ^1^H NMR (DMSO-d6, 400 MHz, δ ppm) δ 7.61–7.57 (m, 2H), 7.48–7.44 (m, 2H), 7.13–7.09 (m, 2H), 6.80–6.76 (m, 2H), 5.86 (s, 1H), 5.44 (s, 1H), 3.66 (s, 1H).). CHN (Elemental analysis) calculated: C, 46.76; H, 2.54; N, 9.62. Found: C, 46.31; H, 2.29; N, 9.77.
4-(4-aminophenyl)-1-(5-(4-bromophenyl)-1,3,4-thiadiazol-2-yl)-3-chloroazetidin-2-one, Mol. Formula C_17_H_12_BrClN_4_OS, Mol.Wt:: 436, Color White, % Yield 71%, M. Pt 212–214 °C, Rf Value 0.7 ** IR (KBr pellets) cm^−1^: (C-H stretching, aromatic), 3099.76, (C=O stretching, azetidine ring), 1717.65 (C=N stretching, imine group), 1638.31, (C=C stretching, aromatic ring), 1595.68, (C-S-C stretching, Thiadiazole ring), 689.88, (C=C stretching, methylene group), 1660, (C-N stretching, imine group), 1228.95, (N=O stretching, Nitro Group), 1514.26, (C-N stretching, Oxadiazole ring), 1170.60, (C-Cl stretching, azetidine ring). 711.65, (C-Br stretching, benzene ring) 581.74. ^1^H NMR (DMSO-d6, 400 MHz, δ ppm) δ 7.61–7.57 (m, 2H), 7.48–7.44 (m, 2H), 7.09–7.05 (m, 2H), 6.62–6.58 (m, 2H), 5.81 (s, 1H), 5.44 (s, 1H), 3.68–3.65 (m, 2H).). CHN (Elemental analysis) calculated: C, 46.86; H, 2.78; N, 12.86. Found: C, 46.23; H, 2.11; N, 12.34.
3-chloro-4-(3,5-dichlorophenyl)-1-(5-(4-methoxyphenyl)-1,3,4-thiadiazol-2-yl)azetidin-2-one, Mol. Formula C_18_H_12_Cl_3_N_3_O_2_S, Mol.Wt:: 441, Color Orange, % Yield 61%, M. Pt 229–231 °C. Rf Value 0.7 *. IR (KBr pellets) cm^−1^: (C-H stretching, aromatic), 3026.81, (C=O stretching, azetidine ring), 1770.90 (C=N stretching, imine group), 1717.54, (C=C stretching, aromatic ring), 1583.98, (C-S-C stretching, Thiadiazole ring), 687.78, (C-N stretching, imine group), 1147.52, (N=O stretching, Nitro Group), 1505.88, (C-N stretching, Oxadiazole ring), 1252.85, (C-Cl stretching, azetidine ring). 614.10, (C-Cl stretching, benzene ring). 748.51. ^1^H NMR (DMSO-d6, 400 MHz, δ ppm) δ 7.59–7.55 (m, 2H), 7.33–7.26 (m, 3H), 7.06–7.02 (m, 2H), 5.94 (s, 1H), 5.44 (s, 1H), 3.82–3.78 (m, 3H).). CHN (Elemental analysis) calculated: C, 49.06; H, 2.74; N, 9.53. Found: C, 48.73; H, 2.14; N, 9.13.
3-chloro-1-(5-(4-methoxyphenyl)-1,3,4-thiadiazol-2-yl)-4-(4-nitrophenyl)azetidin-2-one, Mol. Formula C_18_H_13_ClN_4_O_4_S, Mol.Wt: 417, Color White, % Yield 42%, M. Pt 256–258 °C, Rf Value 0.6 * IR (KBr pellets) cm^−1^: (C-H stretching, aromatic), 3081.85, (C-H stretching, aliphatic), 2927.47, (C=O stretching, azetidine ring), 1724.03, (C=N stretching, imine group), 1642.73, (C=C stretching, aromatic ring), 1625.24, (C-S-C stretching, Thiadiazole ring), 686.12, (C-N stretching, imine group), 1202.49, (N=O stretching, Nitro Group), 1514.13, (C-N stretching, Oxadiazole ring), 1232.21, (C-Cl stretching, azetidine ring). 569.75. ^1^H NMR (DMSO-d6, 400 MHz, δ ppm) δ 8.22–8.18 (m, 2H), 7.59–7.55 (m, 4H), 7.06–7.02 (m, 2H), 6.00 (s, 1H), 5.44 (s, 1H), 3.82–3.78 (m, 3H).). CHN (Elemental analysis) calculated: C, 51.87; H, 3.14; N, 13.44. Found: C, 51.24; H, 3.02; N, 13.09
4-(4-bromophenyl)-3-chloro-1-(5-(4-methoxyphenyl)-1,3,4-thiadiazol-2-yl)azetidin-2-one. Mol. Formula C_18_H_13_BrClN_3_O_2_S, Mol.Wt: 451, Color Brown, % Yield 59%, M. Pt 232–234 °C. Rf Value 0.6 *. IR (KBr pellets) cm^−1^: (C-H stretching, aromatic), 3251.73, (C=O stretching, azetidine ring), 1691.23, (C=N stretching, imine group), 1652.07, (C=C stretching, aromatic ring), 1593.04, (C-S-C stretching, Thiadiazole ring), 690.03, (C-N stretching, imine group), 1159.74, (N=O stretching, Nitro Group), 1444.77, (C-N stretching, Oxadiazole ring), 1333.87, (C-Cl stretching, azetidine ring). 682.38, (C-Br stretching, benzene ring). 539.20. ^1^H NMR (DMSO-d6, 400 MHz, δ ppm) δ 7.59–7.55 (m, 2H), 7.49–7.45 (m, 2H), 7.22–7.18 (m, 2H), 7.06–7.02 (m, 2H), 5.89 (s, 1H), 5.44 (s, 1H), 3.82–3.78 (m, 3H). CHN (Elemental analysis) calculated: C, 47.97; H, 2.91; N, 9.32. Found: C, 47.23; H, 2.11; N, 9.05.
3-chloro-1-(5-(4-chlorophenyl)-1,3,4-thiadiazol-2-yl)-4-(4-nitrophenyl)azetidin-2-one.Mol. Formula C_17_H_10_Cl_2_N_4_O_3_S, Mol. Wt: 421, Color Light red, % Yield 43%, M. Pt 199–201 °C, Rf Value 0.7 *. IR (KBr pellets) cm^−1^: (C-H stretching, aromatic), 3056.78, (C=O stretching, azetidine ring), 1684.47 (C=N stretching, imine group), 1579.22, (C=C stretching, aromatic ring), 1517, (C-S-C stretching, Thiadiazole ring), 633.15, (C=C stretching, methylene group), 1660, (C-N stretching, imine group), (N=O stretching, Nitro Group), 1419.43, (C-N stretching, Oxadiazole ring), 1296.91 (C-Cl stretching, azetidine ring). 766.98, (C-Cl stretching, benzene ring). 823.45. ^1^H NMR (DMSO-d6, 400 MHz, δ ppm) δ 8.22–8.18 (m, 2H), 7.59–7.51 (m, 4H), 7.46–7.42 (m, 2H), 5.99 (s, 1H), 5.44 (s, 1H)). CHN (Elemental analysis) calculated: C, 48.47; H, 2.39; N, 13.30. Found: C, 48.22; H, 2.01; N, 13.11.
1-(5-(4-bromophenyl)-1,3,4-thiadiazol-2-yl)-3-chloro-4-(4-chlorophenyl)azetidin-2-one, Mol. Formula C_17_H_10_BrCl_2_N_3_OS, Mol. Wt: 455, Color Brownish, % Yield 43%, M. Pt 240–242 °C, Rf Value 0.5 *. IR (KBr pellets) cm^−1^: (C-H str, aromatic), 3088.54 (C=O stretching, azetidine ring), 1710.74 (C=N stretching, imine group), 1673.39, (C=C stretching, aromatic ring), (C-S-C stretching, Thiadiazole ring), 689.16, (C-N stretching, imine group), 1278.44, (N=O stretching, Nitro Group), 1543.61, (C-N stretching, Oxadiazole ring), 1175.76, (C-Cl stretching, azetidine ring). 616.78, (C-Br stretching, benzene ring). 519.45, (C-Cl stretching, benzene ring). 759.66. ^1^H NMR (DMSO-d6, 400 MHz, δ ppm) δ 7.61–7.57 (m, 2H), 7.48–7.44 (m, 2H), 7.34–7.30 (m, 2H), 7.24–7.20 (m, 2H), 5.93 (s, 1H), 5.44 (s, 1H)). CHN (Elemental analysis) calculated: C, 44.86; H, 2.21; N, 9.23. Found: C, 44.02; H, 2.11; N, 9.01.

## Data Availability

Data is contained within the article.
